# Supplementary material for: CEO traits, dynamic compensation and capital structure
Source: PLoS One. 2019 Feb 13;14(2):e0211422. doi: 10.1371/journal.pone.0211422 (PMC6373916; doi:10.1371/journal.pone.0211422)
Supplement: S1 File — (ZIP) [file pone.0211422.s001.zip › CEO traits, dynamic compensation and capital structure/CEO traits, dynamic compensation and capital structure.pdf]

CEO traits, dynamic compensation and capital structure

Wei Ye [1,*], Yong Zhang [2],

1 School of Economics and Trade, Hunan University, Changsha, China
2 College of Mathematics and statistics, Jishou University, Jishou, China

These authors contributed equally to this work.
* Corresponding author: 18973108139@189.cn

Abstract

This paper studies the impact of managerial traits, i.e. optimism, confidence and risk aversion, on capital structure using a principle-agent framework. We discover that optimistic manager perceives equity as more undervalued than debt, while, confident manager perceives debt as more undervalued than equity. We also find that there exists the level of risk aversion eliminating the impact of optimism and confidence on the leverage. Furthermore, in contrast to rational manager, the optimistic/confident manger has higher level of effort. And then, the increasing in risk aversion reduces the level of effort. Our results are in line with some empirical findings.

Introduction

How managerial traits affect a corporation's capital structure is an important issue in the corporate finance literature. An optimistic manager overestimates the growth rate of earnings, while a confident manager underestimates the riskiness of earnings. In other words, optimism creates upward bias regarding the mean of the distribution, while confidence creates upward bias regarding its precision. Researchers have extended the psychology literature to behavioral corporate finance and found that managers, as a special group, are more likely to exhibit optimism than ordinary people ([1]). [2] shows that some managers are confident.

[3–5] empirically find that a confident manager perceives firm value to be undervalued by the market. [6] develops a two-period model to obtain these results. [7] considers a continuous model to derive partially empirical results. For example, [7] demonstrates in his model that managerial overconfidence is independent of the investment value, equity is overvalued by the market and an optimistic manager need not follow pecking order theory. These results obtained by [7] are not consistent with some empirical findings, which may be why his model exhibits problems. His model also neglects certain factors such as managerial risk aversion.

To address these and related questions, this paper examines managerial risk aversion and incorporates well-documented managerial traits into a model of dynamic compensation and capital structure. The analysis in the paper extends the study of capital structure to include managerial optimism and confidence. We introduce managerial risk aversion to examine how it affects capital structure and related questions.

Our paper examines capital structure through managerial optimism, confidence and risk aversion in a structural framework. In particular, we extend the model of dynamic compensation and capital structure developed by [8] by incorporating

managerial traits. We also follow [7] and [9]. Our paper differs from their models in two ways. First, we extend the model of dynamic compensation by introducing managerial traits and study a behavioral finance problem. Second, [7] does not consider managerial risk aversion and compensation, whereas we study the interaction of these factors and capital structure.

Our paper provides several important results. First, we show that an optimistic/confident manager perceives debt as being undervalued by the market. The result is exactly the same as that in [7]. We also show that an optimistic/confident manager perceives equity value as being undervalued by the market and unleveraged firm value as being undervalued by the market, which are contrary to the findings in [7]. However, they are consistent with [3, 6, 10]. In this paper, an optimistic manager follows a pecking order, which is not consistent with [7]. Managerial risk aversion mitigates a manager's bias. In other words, the risk aversion reduces managerial incentives to follow a pecking order.

Second, managerial optimism/confidence has a positive effect on the optimal coupon, as in [7]. However, we also have that the optimistic manager's risk aversion has a convex effect on the optimal coupon, while the risk aversion of the confident manager has an negative effect on the optimal coupon. Third, managerial optimism/confidence has a positive effect on actual leverage, which is consistent with [7, 9–11]. An optimistic manager underestimates leverage, as in [7]. However, a less risk-averse and confident manager and a highly risk-averse and confident manager overestimate leverage; a manager with intermediate risk-aversion and confidence overestimates leverage. These results are not consistent with [7]. Interestingly, here, there exists a level of risk aversion that eliminates bias regarding leverage. Moreover, managerial confidence has a positive effect on the owner's bankruptcy level, which is consistent with [7]. However, managerial optimism has an ambiguous effect on the owner's bankruptcy level, and this effect is related to the level of risk aversion. Risk aversion has a convex effect on the owner's bankruptcy level. An optimistic manager underestimates the bankruptcy level, as in [7]. Confident managers with less risk aversion underestimate the bankruptcy level, whereas a confident manager with higher risk aversion overestimates the bankruptcy level. An optimistic/confident manager underestimates the credit spread, which echos the result in [12]. Risk aversion has a convex effect on the credit spread. Finally, relative to a rational manager, an optimistic/confident manger exerts a higher level of effort. This result is consistent with [13–16]. Risk aversion has a negative effect on effort.

The structure of the article is as follows. Section 2 lays out the capital structure framework with managerial traits and dynamic compensation. Section 3 analyzes some analytical and numerical results. Section 4 concludes the article.

The model

Following [8], we assume that a firm hires a manager to operate its business, for which the cash flow X per unit of time depends on its manager's unobservable effort level $a_t \in [0, \overline{a}]$ for $t \geq 0$ and some upper bound $\overline{a} > 0$ according to the following equation:

$$dX_t = (\mu + a_t)X_t dt + \sigma X_t dB_t, \tag{1}$$

where μ is the baseline growth level, $\sigma > 0$ is the instantaneous standard deviation, and dB_t represents the increment of a standard Brownian motion. The manager has so-called managerial traits, meaning that he is optimistic, confident and risk-averse. Specifically, we assume that the manager has CARA instantaneous consumption utility, overestimates the growth level and takes it to be $\mu' > \mu$ while he underestimates the instantaneous standard deviation and takes it to be $\sigma' < \sigma$.

The manager can borrow and save at the risk-free rate r in his personal savings account to smooth his consumption. The account balance, as well as the manager's actual consumption, is unobservable to shareholders, and the following contract is therefore signed.

Contracting problem and solution When a manager is hired, an employment contract $\Pi = \{c; a\}$ is signed between the manager and shareholders that specifies the manager's recommended consumption process c and recommended effort process a. The process c can also be interpreted as the wage process. Both elements are naturally adapted to the filtration generated by X. In other words, they are functions of the managerial performance history.

Following [8], we assume that all agents discount future cash flows or utility at the constant risk-free interest rate r. Given a contract $\Pi = \{c; a\}$, the manager chooses his consumption rate $\hat{c}$ and effort level $\hat{a}$ to solve the following optimization problem:

$$V(\Pi, s, x) = \max_{\{\hat{c}_t, \hat{a}_t\}} \mathbb{E}\left[\int_0^\infty e^{-rt} u(\hat{c}_t, \hat{a}_t) dt | S_0 = s,\ X_0 = x\right] \tag{2}$$

subject to

$$dS_t = rS_t dt + (c_t - \hat{c}_t) dt, \quad dX_t = (\mu' + \hat{a}_t) X_t dt + \sigma' X_t dB_t,$$

where process S represents the manager's savings account balance, the instantaneous utility function $u(z, y) = -\frac{1}{\gamma} e^{-\gamma(z - g(x,y))}$ for $(z, y) \in \mathbb{R}^+ \times [0, \overline{a}]$, γ is the managerial risk-aversion index and the managerial effort cost takes the form $g(x, y) = \frac{\theta}{2} y^2 x$ for some constant $\theta > 0$.

We suppose that shareholders know the managerial traits in advance and the manager has a time-0 outside option with a value of v_0. Because the manager's decisions on his effort, consumption and saving are unobservable, shareholders therefore need to specify a contract $\Pi = \{c; a\}$, which solves

$$J^U(x, v_0) \equiv \max_{\Pi} \mathbb{E}\left[\int_0^\infty e^{-rt} (X_t - c_t) dt | X_0 = x\right] \tag{3}$$

subject to $V(\Pi) \geq v_0$.

Definition 0.1 *A contract $\Pi = \{c; a\}$ is incentive compatible and no savings if the solution to the optimization problem (2) is $\{c; a\}$.*

According to [8], without loss of generality, we consider only the incentive-compatible and no-savings contracts.

Given the contract $\Pi = \{c; a\}$, the manager's continuation value is defined by

$$\mathbb{E}\left[\int_t^\infty e^{-r(s-t)} u(c_s, a_s) ds | S_t = s,\ X_t = x\right], \tag{4}$$

which is equal to $V(\Pi, s, x)$ thanks to (2) and the time-homogeneous system we discuss here. Let $V_t \equiv V(\Pi, S_t, X_t)$, and then by the martingale representation theorem, (4) implies that the manager's continuation value evolves as

$$dV_t = rV_t dt - u(c_t, a_t) dt + \beta_t(-\gamma r V_t)[dX_t - (\mu' + a_t) X_t dt] \tag{5}$$

where β is a progressively measurable process. Due to the CARA utility assumption, the manager's decision on his effort is independent of his savings account balance. Thanks to [8], we further have

$$V(\Pi, S_t, X_t) = V_t(\Pi, 0, X_t) e^{-\gamma r S_t} \tag{6}$$

The intuition behind the result is that the CARA manager has no wealth effect. The CARA manager derive the extra savings S, and his new optimal policy is to make the optimal consumption-effort policy without savings but to consume an extra rS more for all future date $s \geq t$.

Therefore, for simplicity, we assume that the manager's initial wealth is 0 in the following text. Letting $V_t \equiv V(\Pi, 0, X_t)$, we conclude that

$$dV_t = -\gamma r\theta a_t \sigma' X_t V_t dB_t, \tag{7}$$

if contract Π is incentive compatible and no savings. For a proof of similar form, please refer to [8].

Pricing the firm's securities and capital structure

In this subsection, we give the the securities value of firm and the capital structure.

Given the state variables x and V, the shareholders' value function is

$$J^U(x, V) = \max E[\int_t^{\infty} e^{-r(s-t)}(X_s - c_s)|X_t = x], \tag{8}$$

s.t. $V_t(\Pi, x) = V$.

Using the HJB equation for the shareholders' problem in (8), we have

$$rJ^U(x, V) = max_{a\in[0,\bar{a}]}\{x - c + (\mu + a)xJ^U_x + \frac{1}{2}(\sigma x)^2 J^U_{xx} - \gamma r\theta a\sigma'\sigma V_t x^2 J^U_{xV} + \frac{1}{2}(\gamma r\theta a\sigma' V x)^2 J^U_{VV}\}, \tag{9}$$

The absence of any wealth effect, thanks to the CARA preferences, leads to a guess of $J^U(x, V) = f(x) + \frac{1}{\gamma r}\ln(-\gamma rV)$, so we have the unlevered firm value in the following theorem.

Theorem 0.1 *The unlevered firm value solves the equation*

$$rf(x) = \max_{a\in[0,\bar{a}]}\{x + (\mu + a)xf_x + \frac{1}{2}(\sigma x)^2 f_{xx} - \frac{\theta}{2}a^2 x - \frac{1}{2}\gamma r(\theta a\sigma' x)^2\}, \tag{10}$$

and the boundary condition $f(0) = 0$,$\lim_{x\to\infty} f_x = \frac{1}{r-\mu}$

The optimal effort is

$$a^*_t = min(\frac{f_x}{\theta(1 + \theta\gamma r\sigma'^2 x)}, \bar{a}). \tag{11}$$

The proof of the result is similar to [8]. The result provides some implications. First, when x is enough large, we have $f_x = \frac{1}{r-\mu}$. It implies that the marginal value of equity in larger firm is not affected by manager behavior. Second, the manager's effort is affected by manager's risk aversion, the effort cost and the volatility of cash flow.

Next, we consider the leverage firm. The capital structure consists of equity and debt. The firm pays fixed coupon rate C to debtholder before default, and get the remaining firm value at default. The shareholder derive the profit flow $x - (1 - \tau C)$ when the firm is alive, and then get nothing once default. where τ denotes the marginal tax rate. Let x_b denote the default threshold. Following [8], we derive the following result.

Theorem 0.2 *The equity value satisfies the following equation*

$$rE = \max_{a\in[0,\bar{a}]}\{x - (1 - \tau)C + (\mu + a)xE_x + \frac{1}{2}(\sigma x)^2 E_{xx} - \frac{\theta}{2}a^2 x - \frac{1}{2}\gamma r(\theta a\sigma' x)^2\}, \tag{12}$$

and the boundary condition $E(x_b) = 0$, $\lim_{x\to\infty} E(x) = \frac{x}{r-\mu} + \frac{1}{2(r-\mu)^2\theta^2\gamma r\sigma'^2} - \frac{(1-\tau)C}{r}$, *and the smooth-pasting condition* $E_x(x_b) = 0$. *The optimal effort is*

$$a_t^* = min(\frac{E_x}{\theta(1+\theta\gamma r\sigma'^2 x)}, \bar{a}). \quad (13)$$

The debt value satisfies

$$rD = C + (\mu + a^*)xD_x + \frac{1}{2}(\sigma x)^2 D_{xx} \quad (14)$$

the boundary condition $D(x_b) = (1-\alpha)f(x_b)$ *and* $\lim_{x\to\infty} D(x) = \frac{C}{r}$

The optimal coupon C^* *satisfies*

$$C^* = \arg\max_C (E + D). \quad (15)$$

Numerical results and discussion

Baseline case parameters

In our model, we adopt the following baseline parameter values. Volatility rate $\sigma = 25\%$, interest rate $r = 5\%$, bankruptcy cost $\alpha = 25\%$ and tax rate $\tau = 20\%$. Risk aversion $\gamma = 5$, the growth parameter $\mu = -0.5\%$ and $\bar{a} = 5\%$, which are set as in [8]. The effort cost $\theta = 35$, the initial cash flow $x = 50$.

In our numerical analysis, we follow [7]. An irrational manager believes that the shareholders trust her/his financing decisions made based on her/his beliefs $b' = \{\mu', \sigma'\}$. Thus, from the manager's perspective, shareholder beliefs are consistent with the irrational manager's beliefs. In the table below that illustrates the manager's perspective, we give quantities based on the irrational manager's beliefs b', while, in the table that illustrates the market's perspective, we present quantities based on the market's beliefs $b = \{\mu, \sigma\}$, where the debt coupons are chosen by the irrational manager to maximize firm value.

Table 1. Managerial beliefs ($\gamma = 3$).

	U	V	L	C	D	E	x_b	a^*	CR
$\mu = -0.5\%$	923.48	1247.3	48.72%	33.79	607.67	639.63	10.41	0.0296	56
$\mu' = -0.25\%$	968.18	1323.4	49.65%	36.35	657.14	666.29	10.69	0.0310	53
$\mu' = 0\%$	1017.4	1408.5	50.83%	39.44	715.94	692.53	10.07	0.0325	51
$\sigma = 25\%$	923.48	1247.3	48.72%	33.79	607.67	639.63	10.41	0.0296	56
$\sigma' = 22.5\%$	926.86	1331.1	54.77%	39.67	729.07	602.05	12.79	0.0362	44
$\sigma' = 20\%$	931.58	1444.3	61.21%	47.14	884.05	560.29	15.78	0.0456	33

Table 2. Market beliefs ($\gamma = 3$).

	U	V	L	C	D	E	x_b	a^*	CR
$\mu = -0.5\%$	923.48	1247.3	48.72%	33.79	607.67	639.63	10.41	0.0296	56
$\mu' = -0.25\%$	923.48	1246.3	51.51%	36.35	641.94	604.37	11.47	0.0294	66
$\mu' = 0\%$	923.48	1242.2	54.68%	39.44	679.23	563.00	12.76	0.0291	81
$\sigma = 25\%$	923.48	1247.3	48.72%	33.79	607.67	639.63	10.41	0.0296	56
$\sigma' = 22.5\%$	923.48	1241.8	54.90%	39.67	681.78	559.98	12.85	0.0291	82
$\sigma' = 20\%$	923.48	1220.0	61.73%	47.14	753.09	466.91	15.94	0.0281	126

Table 3. Managerial beliefs ($\gamma = 5$).

	U	V	L	C	D	E	x_b	a^*	CR
$\mu = -0.5\%$	917.73	1133.67	39.35%	24.50	446.05	687.62	7.98	0.0182	49
$\mu' = -0.25\%$	961.86	1199.2	40.60%	26.65	486.91	712.25	8.33	0.0190	47
$\mu' = 0\%$	1010.40	1272.20	42.05%	29.20	534.95	737.25	8.75	0.0199	46
$\sigma = 25\%$	917.73	1133.67	39.35%	24.50	446.05	687.62	7.98	0.0182	49
$\sigma' = 22.5\%$	919.75	1190.2	45.59%	29.25	542.66	647.55	10.08	0.0223	39
$\sigma' = 20\%$	922.58	1268.40	53.48%	36.02	678.36	590.06	13.13	0.0279	31

Table 4. Market beliefs ($\gamma = 5$).

	U	V	L	C	D	E	x_b	a^*	CR
$\mu = -0.5\%$	917.73	1133.67	39.35%	24.50	446.05	687.62	7.98	0.0182	49
$\mu' = -0.25\%$	917.73	1133.4	41.98%	26.65	475.84	657.56	8.87	0.0181	60
$\mu' = 0\%$	917.73	1132.40	44.99%	29.20	509.45	622.98	9.91	0.0179	73
$\sigma = 25\%$	917.73	1133.67	39.35%	24.50	446.05	687.62	7.98	0.0182	49
$\sigma' = 22.5\%$	917.73	1132.4	45.04%	29.25	510.08	622.31	9.93	0.0179	73
$\sigma' = 20\%$	917.73	1126.6	52.43%	36.02	590.72	535.88	12.67	0.0175	110

Table 5. Managerial beliefs ($\gamma = 7$).

	U	V	L	C	D	E	x_b	a^*	CR
$\mu = -0.5\%$	915.26	1082.8	43.64%	27.68	472.53	610.25	9.82	0.0129	86
$\mu' = -0.25\%$	959.15	1142.5	43.93%	29.10	501.93	640.56	9.92	0.0135	80
$\mu' = 0\%$	1007.5	1208.9	43.40%	29.93	524.64	684.28	9.72	0.0142	70
$\sigma = 25\%$	915.26	1082.8	43.64%	27.68	472.53	610.25	9.82	0.0129	86
$\sigma' = 22.5\%$	916.71	1123.3	45.39%	28.47	509.82	613.49	10.55	0.0159	58
$\sigma' = 20\%$	918.73	1181.2	48.71%	30.83	575.32	605.91	11.85	0.0202	36

Table 6. Market beliefs ($\gamma = 7$).

	U	V	L	C	D	E	x_b	a^*	CR
$\mu = -0.5\%$	915.26	1082.8	43.64%	27.68	472.53	610.25	9.82	0.0129	86
$\mu' = -0.25\%$	915.26	1082.7	45.32%	29.10	490.64	592.04	10.40	0.0128	93
$\mu' = 0\%$	915.26	1082.6	46.28%	29.93	500.99	581.57	10.73	0.0128	97
$\sigma = 25\%$	915.26	1082.8	43.64%	27.68	472.53	610.25	9.82	0.0129	86
$\sigma' = 22.5\%$	915.26	1082.7	44.58%	28.47	482.68	600.06	10.15	0.0128	90
$\sigma' = 20\%$	915.26	1082.3	50.83%	30.83	511.99	570.34	10.09	0.0127	102

Pecking order theory and managerial traits

Result 1 *An optimistic/confident manager perceives debt and equity as being undervalued. An optimistic manager perceives equity as being more undervalued than debt, while a confident manager perceives debt as being more undervalued than equity. Managerial risk aversion mitigates the manager's bias.*

In tables 1 and 2, on unlevered firm value, the optimistic manager ($\mu' = 0\%$) believes that the firm value is undervalued by 93.92 and the confident manager ($\sigma' = 20\%$) believes that the firm value is undervalued by 8.1. For a levered firm, the optimistic manager ($\mu' = 0$) believes that the debt value is undervalued by 36.71 and that the equity value is undervalued by 129.53. The confident manager ($\sigma' = 0.2$) believes that debt value is undervalued by 130.96 and that the equity value is undervalued by 94.38. Thus, an optimistic/confident manager believes that the value of the firm's securities is undervalued by the market. Because the

optimistic/confident manager prefers to more efforts, and then increase the growth rate of cash flow. This result is consistent with [4,6,10]. However, [7] indicates that an optimistic/confident manager believes that the equity value is overvalued by the market, while a confident manager believes that the unlevered firm value is consistent with market beliefs. His result is not consistent with ours. [7] does not account for managerial risk aversion, whereas we incorporate it into the pricing.

In tables 3-6, when $\gamma = 5(7)$ and the manager is optimistic ($\mu' = 0\%$), for the unlevered firm, the value is undervalued by 92.67 (92.24). For the levered firm, the debt value is undervalued by 25.50 (23.65), and the equity value is undervalued by 114.27 (102.71). Thus, the optimistic manager's level of bias has a negative relationship with risk aversion. When $\gamma = 5(7)$ and the manager is confident ($\sigma' = 20\%$), for the unlevered firm, the value is undervalued by 4.85 (3.47). For the levered firm, the debt value is undervalued by 87.64 (63.33), and the equity value is undervalued by 54.18(35.57). Thus, the confident manager's level of bias has a negative relationship with risk aversion. The intuition behind these results is that risk aversion implies wariness, which is opposite to optimism and confidence.

The optimistic manager perceives equity as being more undervalued than debt. Thus, the optimistic manager prefers the debt financing to equity financing; this follows pecking order theory and is consistent with [12]. By contrast, the confident manager perceives debt as being more undervalued than equity. In contrast to previous contributions, this case need not follow pecking order theory, which is consistent with [7].The intuition behind the results is that the endogenous effort is derive by maximize the equity value. Optimism only increases the growth rate of cash flow. Therefore, the optimistic manager perceives equity as more undervalued than debt. On the contrary, confidence both increases the growth rate and decreases the volatility of cash flow. Because of risk aversion, the the confident manager prefers the equity financing to debt financing.

Capital structure and managerial traits

Optimal coupon and managerial traits

Result 2 *The manager's optimism/confidence has a positive effect on the optimal coupon. The risk aversion of an optimistic manager has a convex effect on the optimal coupon, whereas the risk aversion of the confident manager has an negative effect on the optimal coupon.*

In table 3 and table 4, when the optimistic manager's belief is $\mu' = -0.25\%(0\%)$, the level of the optimal coupon is 26.65 (29.20). When the confident manager's belief is $\sigma' = 22.5\%(20\%)$, the level of the optimal coupon is 29.25(36.02). This result is consistent with [7,9]. When the manager is optimistic ($\mu' = (0\%)$), the optimal coupon is 39.44(29.20/29.93) with risk aversion $\gamma = 3(5/7)$; when the manager is optimistic ($\sigma' = 20\%$), the optimal coupon is 47.14(36.02/30.83) with risk aversion $\gamma = 3(5/7)$. When the risk aversion of the optimistic manager is relatively high or low, the coupon is higher; when his risk aversion is intermediate, the optimal coupon is smaller. By contrast, the optimal coupon decreases with the risk aversion of a confident manager.

Leverage and managerial traits

Result 3 *The manager's optimism/confidence has a positive effect on the actual leverage, The optimistic manager underestimates the leverage. A confident manager with low risk-aversion or high risk-aversion results in overestimates of leverage, while, a confident manager with intermediate risk-aversion leads to overestimated leverage.*

Therefore, there exists a level of risk aversion that eliminates all bias regarding leverage.

In table 3 and table 4, when $\mu' = -0.25\%(0\%)$, the actual leverage is 41.98% (44.99%); when $\sigma' = 22.5\%(20\%)$, the actual leverage is 45.04% (52.43%). This is consistent with [7]. When risk aversion $\gamma = 3(5/7)$, the optimistic manager's ($\mu' = 0\%$) perceived leverage is 50.83% (42.05%/43.40%), whereas the actual leverage is 54.68%(44.99%/46.28%). On the one hand, the risk aversion of an optimistic manager has a convex effect on the perceived (actual) leverage; on the other hand, the optimistic manager underestimates the leverage, and his risk aversion mitigates such bias. When risk aversion $\gamma = 3(5/7)$, the confident manager's ($\sigma' = 20\%$) perceived leverage is 61.21% (53.48%/48.71%), whereas the actual leverage is 61.73%(52.43%/50.83%). The case of a confident manager offers some interesting conclusions. First, risk aversion has a negative effect on the perceived (actual) leverage. Second, the less risk averse and confident manager or the more risk-averse and confident manager underestimates the leverage, whereas a confident manager with intermediate risk aversion overestimates the leverage. Moreover, from the second conclusion, there exists a level of risk aversion that eliminates any bias regarding the leverage.

Bankruptcy level and managerial traits

Result 4 *The manager's optimism has an ambiguous effect on the owner's bankruptcy level, while managerial confidence has a positive effect on the owner's bankruptcy level. Risk aversion has a convex effect on the owner's bankruptcy level. The optimistic manager underestimates the bankruptcy level, the less risk-averse and confident manager underestimates the bankruptcy level, and the more risk-averse and confident manager overestimates the bankruptcy level.*

When the risk aversion $\gamma = 3$, the bankruptcy level of the optimistic manager is 10.69 (10.07). When risk aversion $\gamma = 7$, the bankruptcy level of the optimistic manager is 9.92(9.72). When risk aversion $\gamma = 5$, the bankruptcy level of the optimistic manager is 8.33 (8.75). The manager's optimism has an ambiguous effect on the owner's bankruptcy level. In table 1, the bankruptcy level is 12.79(15.78). Managerial confidence has a positive effect on the owner's bankruptcy level. For an optimistic manager($\mu' = 0\%$), when risk aversion $\gamma = 3(5/7/9)$, the bankruptcy level is 10.07 (8.75/9.72); for a confident manager ($\sigma' = 20\%$), the bankruptcy level is 15.78 (13.13/11.85/12.44). Risk aversion has a convex effect on the owner's bankruptcy level. In table 3 and table 4, when $\mu' = 0\%$, the owner's (market) bankruptcy level is 8.75 (9.91). The optimistic manager underestimates the bankruptcy level. When risk aversion $\gamma = 3$ ($\sigma' = 20\%$), the owner's (market) bankruptcy level is 15.78 (15.94). When risk aversion $\gamma = 5$ ($\sigma' = 20\%$), the owner's (market) bankruptcy level is 13.13 (12.67). The less risk-averse and confident manager underestimates the bankruptcy level, the the more risk-averse and confident manager overestimates the bankruptcy level.

Credit spread and managerial traits

Result 5 *The optimistic/confident manager underestimates the credit spread. Risk aversion has a convex effect on the credit spread.*

In table 4, when $\mu' = -0.25\%(0\%)$, the perceived credit spread is 47bps (46bps), and the actual credit spread is 60bps (73bps); when $\sigma' = 22.5\%(20\%)$, the perceived credit spread is 39bps (31bps), and the actual credit spread is 73bps (110bps). The

optimistic/confident manager underestimates the credit spread. This is consistent with [12]. When $\mu' = -0.25\%$ and risk aversion $\gamma = 3(5/7)$, the actual credit spread is 66bps (60bps/93bps). When $\sigma' = 22.5\%$, risk aversion $\gamma = 3(5/7)$, the actual credit spread is 82bps (73bps/90bps). Risk aversion has a convex effect on the credit spread.

Managerial effort and managerial traits

Result 6 *Relative to a rational manager, an optimistic/confident manger exerts a higher level of effort. Risk aversion has a negative effect on effort.*

In table 3 and table 4, when $\mu' = 0\%$, the optimistic manager's effort is 0.0190, and the rational manager's effort is 0.0179; when $\sigma' = 20\%$, the confident manager's effort is 0.0279, and the rational manager's effort is 0.0175. This is consistent with [14, 15, 17]. When $\mu' = 0\%$, and risk aversion $\gamma = 3(5/7)$, the level of managerial effort is 0.0325 (0.0199/0.0142). When $\sigma' = 20\%$, and risk aversion $\gamma = 3(5/7)$, the level of managerial effort is 0.0456 (0.0279/0.0202). Risk aversion has a negative effect on effort.

Conclusion

This paper examines the impact of managerial optimism, confidence and risk aversion on the capital structure, peaking order theory and managerial welfare. The model developed here demonstrates that an optimistic manager perceives equity as being more undervalued than debt, whereas a confident manager perceives debt as being more undervalued than equity. The manager's optimism has an ambiguous effect on the owner's bankruptcy level. An optimistic/confident manager underestimates the credit spread.

This paper introduces risk aversion into the model. We show that an optimistic manager follows a pecking order, which is consistent with previous empirical results. In this paper, we find that managerial risk aversion has an important impact on the capital structure. Managerial risk aversion mitigates the manager's bias regarding firm value. The risk aversion of the optimistic manager has a convex effect on the optimal coupon. There exists a level of risk aversion that eliminates the bias regarding leverage. Risk aversion has a convex effect on the owner's bankruptcy level. Risk aversion has a convex effect on the credit spread.

From a behavioral finance perspective, our paper examined the effects of managerial traits on managerial effort and welfare. Relative to a rational manager, an optimistic/confident manger exerts a higher level of effort. Risk aversion has a negative effect on effort. In addition, in our model, we consider a capital structure that includes equity and straight bonds. The capital structure can be composed of straight bonds, junk bonds and equity. Our approach may explain why straight bonds are not replaced by junk bonds. [18] consider the impacts of internal financing on investment decisions in the context of managerial traits. Our model makes it possible to study the investment from the perspective of managerial traits when investment costs can be raised through internal cash, equity and bonds. It can also be used to explore whether a firm follows a peaking order. These are promising and significant avenues for future research.

Supporting information

S1 File. CEO traits, dynamic compensation and capital structure.zip

Acknowledgments

The authors wish to thank two anonymous referees for the constructive comments, which greatly helped in improving the paper.

A The proof of Theorem 0.1

In order to the optimality of the agent's consumption-savings policy, his marginal utility from consumption must equal his marginal value of wealth. We derive the following relation:

$$u(c_t, a_t) = rV_t. \tag{16}$$

Since $u(c_t, a_t) = -e^{-\gamma(c_t - g(X_t, a_t))}/\gamma$, and then we have

$$c_t = g(X_t, a_t) - \frac{1}{\gamma}\ln(-\gamma r V_t) \tag{17}$$

We have $J_x = f_x$, $J_{xx} = f_{xx}$, $J_{VV} = -1/(\gamma r V^2)$ and $J_{xV} = 0$. Plugging (17) and the following formulations into (9). We derive the equation (12). By first order condition, we immediately derive the equation (13). Let $f(x) = ax + b$ when $x \to \infty$, by (12) and (13), we get the $a = 1/(r - \mu)$ and $b = 1/(2(r-\mu)^2\theta^2\gamma r \sigma^{'2})$. Therefore, we can derive that $\lim_{x\to\infty} f_x = 1/(r - \mu)$.

B The proof of Theorem 0.2

In spirit of The proof of Theorem 0.1, we can derive the HJB of equity value. Given the implemented effort policy, the debt value satisfies (14). The optimal coupon is derived by maximizing firm total value, as is given by (15). The intuition behind the result is that the two opposite effect of debt. On the one hand, the debt can bring tax shield profit and increase the firm value, we call it as the tax shield effect. On the other hand, the debt results in default and causes bankruptcy cost. We call it as bankruptcy effect. The first effect makes firm to issue more debt, however, the second effect incentives firm to issue less debt. As a result, The firm chooses the optimal coupon to maximizing firm total firm.

References

1. Landier A, Thesmar D. Financial contracting with optimistic entrepreneurs. Review of Financial Studies. 2009; 22(1): 117–150.

2. Bernardo AE, Welch I. On the evolution of overconfidence and entrepreneurs. Journal of Economics & Management Strategy. 2001; 10(3): 301–330.

3. Malmendier U, Tate G. Ceo overconfidence and corporate investment. The Journal of Finance. 2005: 60(6): 2661–2700.

4. Malmendier U, Tate G, Yan J. Corporate financial policies with overconfident managers. Tech. rep., National Bureau of Economic Research. 2007.

5. Lin Y, Hu S, Chen M. Testing pecking order prediction from the viewpoint of managerial optimism: Some empirical evidence from taiwan. Pacific-Basin Finance Journal. 2008; 16(1): 160–181.

6. Heaton JB. Managerial optimism and corporate finance. Financial management. 2002; 31(2): 33–45.

7. Hackbarth D. Managerial traits and capital structure decisions. Journal of Financial and Quantitative Analysis. 2008; 43(4): 843–881.

8. He Z. A model of dynamic compensation and capital structure. Journal of Financial Economics. 2011; 100(2): 351–366.

9. Hackbarth D. Determinants of corporate borrowing: A behavioral perspective. Journal of Corporate Finance. 2009; 15(4): 389–411.

10. Malmendier U, Tate G, Yan J. Overconfidence and early-life experiences: the effect of managerial traits on corporate financial policies. The Journal of Finance. 2011; 66(5): 1687–1733.

11. Yu M, Xia X, Zou Z. The relationship between managers' overconfidence and enterprises' radical behavior in incurring debts. Management world [in Chinese]. 2006; 8: 358–379.

12. Adam T, Burg V, Scheinert T, Streitz D. Managerial optimism and debt contract design. Working Paper, 2014.

13. Gervais S, Heaton J, Odean T. The positive role of overconfidence and optimism in investment policy. Working paper. 2002.

14. De la Rosa LE. Overconfidence and moral hazard. Games and Economic Behavior. 2011; 73(2): 429–451.

15. Chen Q, Li X. A model on agent-principal relationship with overconfident agents. Journal of Management Engineering [in Chinese]. 2007; 1: 110–116.

16. Gervais S, Heaton J, Odean T. Overconfidence, investment policy, and manager welfare. Working paper. 2007.

17. Ye, B., Yuan, J., 2008. Firm value, managerial confidence, and investments: The case of china. Journal of Leadership Studies 2 (3), 26–36.

18. Kamoto S. Impacts of internal financing on investment decisions by optimistic and overconfident managers. European Financial Management. 2014; 20(1): 107–125.
